# Knee movement patterns of injured and uninjured adolescent basketball players when landing from a jump: A case-control study

**DOI:** 10.1186/1471-2474-7-22

**Published:** 2006-03-07

**Authors:** Quinette Louw, Karen Grimmer, Christopher Vaughan

**Affiliations:** 1 Department of Physiotherapy, Stellenbosch University, P O Box 19063, Tygerberg, 7505, South Africa; 2 Division of Health Sciences, University of South Australia, City East Campus, North Tce, Adelaide 5000, Australia; 3 Department of Human Biology, University of Cape Town, South Africa

## Abstract

**Background:**

A common knee injury mechanism sustained during basketball is landing badly from a jump. Landing is a complex task and requires good coordination, dynamic muscle control and flexibility. For adolescents whose coordination and motor control has not fully matured, landing badly from a jump can present a significant risk for injury. There is currently limited biomechanical information regarding the lower limb kinetics of adolescents when jumping, specifically regarding jump kinematics comparing injured with uninjured adolescents. This study reports on an investigation of biomechanical differences in landing patterns of uninjured and injured adolescent basketball players.

**Methods:**

A matched case-control study design was employed. Twenty-two basketball players aged 14–16 years participated in the study: eleven previously knee-injured and eleven uninjured players matched with cases for age, gender, weight, height and years of play, and playing for the same club. Six high-speed, three-dimensional Vicon 370 cameras (120 Hz), Vicon biomechanical software and SAS Version 8 software were employed to analyse landing patterns when subjects performed a "jump shot". Linear correlations determined functional relationships between the biomechanical performance of lower limb joints, and paired t-tests determined differences between the normalised peak biomechanical parameters.

**Results:**

The average peak vertical ground reaction forces between the cases and controls were similar. The average peak ground reaction forces between the cases and controls were moderately correlated (r = -0.47). The control (uninjured) players had significantly greater hip and knee flexion angles and significantly greater eccentric activity on landing than the uninjured cases (*p *< 0.01).

**Conclusion:**

The findings of the study indicate that players with a history of knee injuries had biomechanically compromised landing techniques when compared with uninjured players matched for gender, age and club. Descriptions (norms) of expected levels of knee control, proprioceptive acuity and eccentric strength relative to landing from a jump, at different ages and physical developmental stages, would assist clinicians and coaches to identify players with inappropriate knee performance comparable to their age or developmental stage.

## Background

Landing from a jump is a complex task, often not well mastered by adolescents or adults [1]. Landing poorly from a jump is a common adolescent sports knee injury mechanism [2]. The prevalence of sports-related knee injuries among adolescents is high [2,3], and knee injuries sustained by adolescents during landing can result in serious outcomes such as lengthy time lost from play, or expensive medical management [3].

Reported adolescent knee injury prevalence ranges between 10%–20% [4-6]. Injury to adolescent knees should be a public health concern because of the increased likelihood of leaving regular physical activity, and developing early osteoarthritis [7]. Regular physical activity is effective in maintaining good health and preventing the onset of chronic lifestyle diseases [8]. Thus attrition from regular exercise puts young people at increased risk of developing lifestyle-related health problems.

Research into knee injury causality, and identification of risk factors that may predispose adolescent basketball players to knee injury, could assist in the development of knee injury-prevention programs [9] to reduce injury prevalence and assist young people to maintain active lifestyles. Although adolescent knee sports-injuries are usually associated with complex intrinsic and extrinsic factors [2,4-9], a better understanding of the biomechanics of jump-landing techniques would provide insights into important knee-injury risk factors that may be preventable. Limited research on knee injury associated with poor landing has identified inefficient lower limb kinetics and kinematics, being female and pubescence as key risk factors [10-19]. Reported lower limb joint kinetics, and kinematics risk factors include high ground reaction forces, high peak knee adduction, abduction and extension moments, landing with the knee more extended and increased tibial-torsional moments [14,15,19-22].

The injury risk factors relating to adolescent sporting participants differ from those of adults [14]. A recent search of MEDLINE, CINAHL, Current Contents and SPORTDiscuss identified 26 relevant studies about sports-landing techniques published between 1990 and 2004. Only six of these studies reported on injured subjects, all of whom were adults [10,11,13,18,19,21]. This highlights the current lack of information on adolescent lower limb kinetics and kinematics.

This paper reports on the findings of a study which examined kinetic and kinematic differences in landing patterns of previously knee-injured and uninjured adolescent basketball players. The hypothesis tested was that, compared with matched controls (non-knee-injured players), injured adolescent basketballers would demonstrate higher vertical ground reaction forces, reduced knee flexion upon landing and reduced negative work at the knee.

## Methods

### Study design and sampling

A case-control study design was employed to identify biomechanical differences in performance related to the effect of previous knee injury. In an earlier survey capturing approximately 70% (N = 458) of the competitive adolescent basketball players in Cape Town [3], we identified a cohort of 97 players who had sustained knee injuries during the 2002 basketball season. Eleven cases for the study reported in this paper were conveniently selected from this previously injured cohort on the basis of appropriate matching with uninjured players by age, gender, weight, height and years of play, and playing for the same club. These young people had previously suffered a knee injury (and no other lower limb joint injuries) while playing basketball during the preceding season, which resulted in them missing one or more basketball playing sessions (training or competition). The knee injury mechanisms included landing badly from a jump (seven players), falling after landing (two players), bumping into another player (one player) and pain related to overuse (one player). The location of the injuries was mostly patellofemoral, as indicated by the area and behaviour of the symptoms (eight players) and minor ligamentous injuries (three players) [23]. None of the players suffered from severe knee instability as a result of the injury, and none had undergone knee operations. None of the injuries had been treated formally with physiotherapy or rehabilitation programs.

At the time of testing, none of the injured players had knee range of motion limitations and from the players' perspectives, their knee injuries had healed. This was indicated by their functional ability to play basketball. Injured players were excluded from the case sample if they had significantly reduced lower limb joint range due to skeletal diseases, congenital deformities or other contractures due to e.g. severe burns. Once the case players were identified, a list of potential control players were identified from the survey cohort, matched for age, gender, weight, height and years of play, and playing for the same club. Control players had never suffered a knee injury or any other lower limb joint injury, and were similarly unaffiliated by skeletal diseases, congenital deformities or other contractures due to e.g. severe burns.

Twenty-two basketball players (11 previously knee-injured and 11 uninjured) aged 14–16 years participated in the study. The sample included four fourteen year old boys (two cases and matched controls), four fifteen year old boys (two cases and matched controls), four sixteen year old boys (two cases and matched controls), four fourteen year old girls (two cases and matched controls), four fifteen year old girls (two cases and matched controls) and two sixteen year old girls (one case and matched control). The average weight of the controls was 538 Newton (N) (SD 96.64) and the cases 560 N (SD 66.93). Average height of the cases was 1.63 m (SD 0.18), and among the controls was 1.64 m (SD 0.73). There were no significant differences in the height and weight between the cases and controls (*p *> 0.05).

### Ethical approval

Ethical approval for the study was obtained from the University of South Australia Human Research Ethics Committee. Parents and subjects gave informed written consent for their child to participate in the study.

### Subjective evaluation of knee complaints

All injured players completed the Hughston Visual Analog Knee Scale to evaluate their knee complaints at the time of testing [24]. This scale contains 28-items that measure signs and symptoms on continuous scales (100 mm long lines with polar descriptors of the two extremes of the symptom experience at each end of the line). Subjects read the description and placed a mark on the line at the point that represented their experience, relative to the two extremes. Responses were converted to a numerical value by measuring from the left starting point to the point where the subject marked the line. The final score was derived by summing the 28 symptom responses of a subject and thereafter the average score for the subject was calculated and indicated as a number out of 100. Scores of 55/100 were considered as the cut-off score for reasonable healing of knee injuries in conjunction with the ability to play basketball of the case subjects [25]. Similar scores were noted after clinical recovery of patient who had knee surgery for patellar tendinosis [25].

### Measurement tools

Six high-speed Vicon 370 cameras (120 Hz) (Oxford Metrics, Oxford, UK) and biomechanical software were used to analyse landing patterns. The six cameras were strategically positioned in the laboratory to ensure that all anatomical markers were captured throughout the trial (see below). A strain gauge 6-channel force plate (AMTI Inc., Newton MA), synchronised with the Vicon System, was used to measure the ground reaction forces.

### Vicon laboratory testing procedures

#### Subject preparation

One researcher (Quinette Louw) took all anthropometric measurements, comprising body mass, height, anterior superior iliac spine breadth, thigh length, mid-thigh circumference, knee diameter, foot length, malleolus height, malleolus width and foot breadth. These measures were required in the analysis for kinetic and kinematic data. As previously reported by Vaughan et al, a Holtain calliper, tape measure and a standard anatomical marker set developed for lower limb testing was used for anthropometric measures [26]. Fifteen retro-reflective markers were applied to the subject's anterior superior iliac spines, sacrum, mid-thigh, knee joint, mid- lateral leg, lateral malleoli, base of the second metatarsal and calcaneous for biomechanical analysis.

#### Trial capture

All subjects performed a standard 10-minute warm-up session consisting of jogging and stretching upper and lower limb muscles.

The subjects performed two practice jumps to familiarise themselves with the laboratory equipment and testing procedures. Thereafter they performed 10 "jump-shots", landing with each foot 5 times on the force plate. These 10 trials were captured on video. Subjects performed barefoot to aid reliability of marker placement and to reduce the effect of differences in footwear between players. A 2 cm soft rubber mat was placed over the force plate to accommodate for the padding usually offered by shoes during landing from a jump, and to prevent pain when landing directly onto the force plate. The choice of landing with the right or left foot on the force plate first was determined by the subject, and no instructions were given by the researcher. Players were given the following standard instructions: "Run forward for three steps and land with one foot on the force plate", "Jump as high as possible and simulate shooting a basketball".

#### Trial data capture

Trial data capture commenced as the subject ran up towards the force plate, and ended after the subject had landed on the force plate. The trial was deemed acceptable if the correct jump was performed and all the retro-reflective markers were tracked on the computer monitor during the landing phase. Subjects had to maintain their balance when landing on the force plate so that they did not fall when landing. Ten trials were processed for each subject.

#### Trial data validation

Intermittent gaps in the trajectory of a selected parameter were filled in by the Workstation program by linear interpolation from the surrounding frames. This interpolation provided a reasonable estimate of the position of the marker during the period it was obscured (Workstation Biomechanical Software by Oxford Metric (Oxford, UK). If the maximum number of gaps to be filled was larger than 10 consecutive frames, then the interpolated points were no longer deemed to be an accurate estimate and the trial was rejected (Workstation Biomechanical Software, Oxford Metric-Oxford, UK). Once the markers were filtered and data gaps filled the trial files were accepted for analysis.

#### Trial data processing

Biomechanical parameters were collected and processed to C3D files with Workstation Biomechanical Software by Oxford Metric (Oxford, UK). The C3D files were further converted to DST files using Rdata2 program by Motion Lab Systems. Processing the raw data files was accomplished by Bodybuilder program by Oxford Metrics. Trials were truncated to highlight the landing phase since this was the major focus of this study. All analyses were based on measurements from the airborne position of the jump shot until after the subject had landed on the force plate. Three-dimensional joint kinematics and force data were obtained from the Vicon system and Vicon software. Moment, power and work values were calculated using well accepted biomechanical techniques [26]. A kinetic mathematical model based on the inverse dynamics approach was applied in Bodybuilder to calculate kinetic values [26]. The total negative work (eccentric action) was obtained by using Gaitlab program by Kiboho Publishers [26]. Total negative work was calculated from the trapezoid integration of the area below the negative portion of the power/time graphs [27,28]. All data was exported as text files for analysis in Excel (Microsoft Corporation).

Data was standardised to allow for comparison between subjects of different sizes. The moment and work data were standardized to body weight and height and expressed as a percentage. The moment data were normalised to body height and weight (hence the unit, % body weight X height) [22]. Impact force was expressed as a component of body weight (hence the unit, X body weight) [22].

### Data analysis

The data from the formerly injured leg of the cases was compared to the same leg of the controls. All subjects were asked to identify their dominant side, however many of the younger players were unable to indicate their dominant leg. This may be because adolescents only become aware of dominance in the later period of their skill development. It may also be due to the dynamic nature of basketball, since players are trained to react equally well with the left and right sides. The same leg of injured and uninjured players was thus compared in all correlation analysis.

SAS Version 8 was used to analyse the data. SAS programs were written to perform the following statistical procedures.

Linear correlations were undertaken to assess the functional relationship between the biomechanical measures taken from the lower limb joints (hip and knee). Correlations were described by r square correlation coefficients and associated p-values. Correlations of hip and knee angles were determined by using the frame at maximum vertical ground reaction force as the reference point i.e. all correlations calculations used the value of the parameter at maximum vertical ground reaction force.

Paired t-tests were applied to determine significant differences between the normalised peak biomechanical parameters, a test which was appropriate as the cases and controls were individually matched. The maximum values of each of the five trials of cases were compared to the maximum values of the same trial values of the controls. Significance levels for all tests were set at *p *< 0.01 to correct for chance findings derived from multiple comparisons.

## Results

### Subjective knee complaint scores according to age and gender

The subjective injury score was analysed by age and gender. Among the males, the fourteen year-olds had the worst injury score, whilst among the females, the 16 year-old females presented with the highest injury score, and they also had the highest injury score compared with all other age and gender groups (Table 1).

### Ground reaction force

The data of the right and left leg trials (n = 220 trials) were analysed and the average peak ground reaction forces of the cases and controls are presented in Table 2. The average peak ground reaction forces between the cases and controls are comparable.

### Maximum ground reaction force correlated with average injury complaint score

Maximum ground reaction force on the injured side of the cases was correlated with the average injury score. A positive correlation (r = 0.49, *p *< 0.01) was found between the maximum ground reaction force and the average injury score of the case subjects. Thus, the worse the injury, the higher the peak ground reaction force.

### Correlation between maximum ground reaction force and knee angles

Peak knee flexion angles were negatively correlated with peak ground reaction force. The trend was consistent across case and control subjects (Table 3).

### Differences in peak vertical ground reaction force

The maximum vertical ground reaction among the cases was 2.5 times body weight and 2.7 times body weight among the controls when performing the jump-shot. This difference was not significantly different (*p *> 0.05).

### Differences in peak hip flexion angles

The average maximum hip flexion jump shot angles among the controls was 53.5 (SD 10.4) and among the cases 47.7 degrees (SD13.6), the difference of approximately six degrees in hip flexion angle being significant (*p *< 0.01).

### Differences in peak knee flexion angles

The control players had significantly deeper knee flexion angles than the cases on landing (*p *< 0.01). The average maximum knee flexion angle of the control players was 66.4 degrees (SD 12.6) and 57.1 degrees (SD 13.3) among the cases.

### Differences in total negative work

Negative work during the landing action after performing the jump-shot is presented in Figure 1. The injured players demonstrated significantly less eccentric activity on landing compared with the uninjured players (*p *< 0.01). The average negative work demonstrated by the injured players was -2.14 J/N.m compared to -3.37 J/N.m by the uninjured players.

The average negative work among the uninjured girls (Figure 2) was -0.89 J/N.m and among the injured girls -2.02 J/N.m (*p *< 0.05). The uninjured boys (Figure 2) demonstrated negative work of about -3.84 J/N.m while the injured boys only demonstrated negative work of -2.26 J/N.m. There was a significant difference in negative work (*p *< 0.05) between the male and female groups since the boys demonstrate greater negative work compared with girls.

### Differences in peak joint moments

Moment data was standardized by height and weight to enable inter-subject comparison. None of the hip and knee maximum joint moments differed significantly between the cases and controls when performing the jump-shot landings (Table 4).

## Discussion

This study provides the first known biomechanical findings comparing previous-knee-injured with never-knee-injured adolescent basketballers' capacity to land from a jump. It adds to the scarce literature regarding recovery of adolescents with knee injuries with respect to motor control, motor planning and proprioception. This study therefore provides important information for coaches, clinicians and players, and suggests a framework within which previously injured players can be rehabilitated in order to prevent injury re-occurrence.

In this study, a key finding was the strong positive correlation between subjective injury score and ground reaction forces. McNair and Marshall [18], in a similar study, noted that not all anterior cruciate ligament -eficient subjects protected their knees when performing a jump landing task. This suggests that players could be taught preventative strategies to protect their injured knees from further injury, and that protective compensatory mechanisms are not automatically employed. These researchers found no differences in kinematic and kinetic measures between injured and uninjured subjects. This is of note as the odds of sustaining a recurrence of an injury to the knee is significantly higher compared with all other joints [29]. It should be noted however that the knee injuries previously sustained by the subjects in this study, were measured by the Hughston Visual Analog Knee Scale, subjectively by questionnaire and physical examination. Diagnostic evaluation such as imaging techniques was not undertaken and this limitation may well have influenced the knee injury classifications.

The study findings highlight that parents, coaches and players should not consider knee injuries to have fully recovered biomechanically, once subjective symptoms, such as pain, improve. Subjective complaints are generally regarded as an indication of the stage of injury healing [30]. Consequently injured players often expect, and coaches expect them, to resume playing basketball based on merely symptomatic improvement and not assessment of functional dynamic knee control. Health promotion programs should educate all key personnel involved in young basketball players' rehabilitation on safe return to sports. This includes sufficient rest post-injury, alternative or safer training methods during the injured stage and ensuring good knee dynamic stability before recommencing basketball post-injury.

The literature suggests that more knee flexion during the landing phase will reduce the chances of injury due to lower ground reaction forces and better shock absorption [22]. The results of this study imply a relationship between peak knee flexion angle and knee injury. However, this was a cross-sectional study that cannot test causation, and is thus not possible to ascertain whether reduced knee flexion when landing, is a cause, or effect of, knee injury.

Previous research has analysed peak ground reaction forces and peak kinematic parameters such as knee flexion angles when considering landing [22]. However the maximum degree of knee flexion at the time of peak ground reaction force may be more important clinically. The instance of maximum ground reaction force angle reflects the point when the knee joint structures are most strained by the impact load. Our data demonstrated a negative correlation between the degree of knee flexion at the time of maximum ground reaction force. More knee flexion at the time of maximum ground reaction force is related to lower peak ground reaction force values. The high correlation between knee angle and maximum ground reaction force suggest that the degree of knee flexion could possibly be one of the most important factors related to reduced impact after landing from a jump. Other factors impacting on this relationship may be ankle control, leg dominance, hip control, trunk stability, take-off and upper limb activity at the time of peak ground reaction force [32]. Therefore, the degree of knee flexion may be the single most important factor related to reduced impact.

On the basis of the findings of this study, preventative programs should not only aim to produce appropriate knee flexion angles during the entire motion (landing from a jump), but also to train athletes about the critical point to flex their knees, which may be at the point of maximum ground reaction force. In practice, this may be difficult to train players to do, as force plates are generally not available at training sessions. The average time from foot contact to maximum ground reaction force is about 0.03 seconds [33]. This reflects a very short time period and the clinical implication is that players should be encouraged to flex their knees on, or soon after, foot contact with the ground, thereby optimising injury prevention.

Of note was the significant difference in negative work found between the injured and uninjured players when landing from a jump. Negative or eccentric muscle work is regarded as an important component in maintaining balance and knee stability when performing precarious movements such as landing from a jump [34]. Joint stability is determined by an interaction of passive restraints produced by the ligaments and other joint structures, joint geometry, friction between cartilage surfaces and stability provided by muscles acting on the joint [35]. Of all these factors the stability provided by the contracting muscles appears to be the most important for knee stability [34]. Eccentric muscle action stabilises the knee joint dynamically as eccentric muscle contractions act to control deceleration of body segments during dynamic tasks [35]. Players who are less skilled in controlling knee flexion during landing may also fatigue faster compared with skilled players [36]. Muscle fatigue not only impairs performance, but also impairs proprioceptive acuity and reduces pre-activation of stabilising muscles [36]. Players with poorer landing mechanics may thus lose their balance leading to twisting movements at the knees or falls that could stress and injure soft tissues, particularly ligamentous structures [36]. Furthermore, players who demonstrate good knee stability will be more capable of flexing their knees adequately to appropriately absorb impact forces when landing due to better proprioceptive ability and consequent neuromuscular control [35].

During eccentric contractions, the force that the muscle can generate is much higher than during concentric contractions, as much lower motor activity is required during concentric contractions [37]. Eccentric muscle contractions are consequently more often related to muscular injury due to high concurrent tension levels in the muscle. The players who demonstrate strength deficits will therefore not be able to tolerate high eccentric forces leaving them vulnerable to not only ligamentous injuries but also muscle or tendon injuries whilst landing from a jump [37].

The differences noted in the landing patterns of injured and uninjured basketball players, indicates that the injured players may have an increased risk to sustaining a knee injury. However, due to the retrospective design of this study, the difference in performance cannot be directly attributed to the knee injury.

## Conclusion

Adolescents who had previously sustained knee injuries demonstrated biomechanically compromised landing mechanics compared with matched never-injured players. Identification of potential injury risk factors for which intervention could occur allow opportunities for clinicians to contribute towards injury prevention. Future studies should prospectively explore the relationship between biomechanical parameters and knee injury occurrence. Norms of expected levels of knee control, proprioceptive acuity and eccentric strength at different ages or developmental stages could aid clinicians and coaches in identifying players who demonstrate appropriate (or inappropriate) control comparable to their age or developmental stage. This paper highlights the need for injury prevention programs based on sound biomechanical analysis as an integral part of adolescent basketball coaching.

## Competing interests

The author(s) declare that they have no competing interests.

## Authors' contributions

Quinette Louw conceived the study, conducted the research and drafted the manuscript.

Karen Grimmer supervised the study, assisted with statistical analysis and drafting the manuscript.

Christopher Vaughan assisted with data collection, data processing and analysis.

## Pre-publication history

The pre-publication history for this paper can be accessed here:


